# Research on the Poisson’s Ratio of Black Phosphorene Nanotubes Under Axial Tension

**DOI:** 10.3390/nano15161259

**Published:** 2025-08-15

**Authors:** Xinjun Tan, Touwen Fan, Kaiwang Zhang

**Affiliations:** 1 School of Physics and Optoelectronics, Xiangtan University, Xiangtan 411105, China; 2 Research Institute of Automobile Parts Technology, Hunan Institute of Technology, Hengyang 421002, China; 3 School of Science, Hunan Institute of Technology, Hengyang 421002, China

**Keywords:** negative Poisson’s ratio, molecular dynamics, black phosphorene nanotubes

## Abstract

In this paper, the Poisson’s ratio of black phosphorene nanotubes was examined through the molecular dynamics simulation method. Our research discovered that for the armchair black phosphorene nanotubes, the radial strain and the wall thickness strain are negatively linearly correlated with the axial strain, and both the radial Poisson’s ratio and the thickness Poisson’s ratio are positive. For the zigzag black phosphorene nanotubes, the wall thickness strain is negatively, linearly correlated with the axial strain, while the radial strain has a cubic polynomial function relationship with the axial strain. The thickness Poisson’s ratio is positive, while the radial Poisson’s ratio is a quantity related to the axial strain. As the axial strain increases, the radial Poisson’s ratio progressively diminishes from a positive value and becomes negative upon reaching a specific critical axial strain threshold. During the tensile deformation along the axial direction of the zigzag black phosphorene nanotubes, the radial strain initially decreases before subsequently increasing. Notably, the diameter of the nanotube may even surpass its initial value, demonstrating a radial expansion in response to axial tension.

## 1. Introduction

Poisson’s ratio is a basic mechanical property of materials, which relates the resulting transverse strain to the applied axial strain. It refers to the negative ratio of the lateral normal strain to the longitudinal normal strain when a material is subjected to longitudinal tension or compression. According to the theory of continuum elasticity, it is feasible for a material to have a negative Poisson’s ratio (NPR). If a certain material has an NPR, it implies that when this material is under longitudinal tension, it will expand in a certain lateral direction. Nevertheless, in nature, nearly all natural materials have a positive Poisson’s ratio (PPR). Ever since the discovery of the NPR property in foam-structured materials by scholars in 1987 [1], the research on NPR materials has aroused people’s attention. NPR materials typically possess enhanced toughness and shear resistance, as well as enhanced sound absorption and vibration damping, and have been applied in fields such as medical treatment, fasteners, high-strength composite materials, sensors, biological tissue engineering, textile materials, protective engineering, etc. [2,3,4,5,6,7,8,9].

For a considerable period, materials with NPR were all artificially fabricated materials featuring hinge-like structures [5,8,10,11,12]. In 2015, Jiang [2] discovered through first-principles calculations that single-layer black phosphorene (BP) exhibits an NPR in the direction perpendicular to the BP surface when stretched along the armchair direction. This finding was experimentally verified in 2016 [13]. This was the first time that scholars discovered a natural material with a negative Poisson’s ratio, and this discovery has sparked significant interest among numerous scholars in materials with NPR. Existing research indicates that two-dimensional materials with wrinkles similar to those of black phosphene all possess the characteristic of NPR [3,4,6,9,14,15,16,17,18,19,20,21,22,23,24,25,26,27]. Additionally, some scholars contend that two-dimensional materials with honeycomb structures will all display NPR properties under appropriate strains [19]. Considering that BP exhibits a negative Poisson’s ratio in the vertical plane direction, we are curious about the result of the Poisson’s ratio in the radial direction after curling BP into black phosphorus nanotubes (BPNTs). The structural stability of BPNTs is related to their radius and environmental temperature; the larger the diameter of BPNTs, the smaller the strain energy. Theoretical research indicates that at a specific ambient temperature, when the radius of the BPNTs exceeds a certain critical value, the structural stability can be maintained [28,29,30,31,32]. Phonon spectrum calculations indicate that BPNTs exhibit stability and possess physical properties similar to that of bulk BP [29,31,33,34]. Although BPNTs have not yet been successfully prepared in the laboratory, this does not prevent scholars from exploring and researching them. Through molecular dynamics simulation and calculation methods, scholars have pointed out that phosphorene can be wound around carbon nanotubes to obtain BPNTs [35,36,37,38]. Currently, in experiments, phosphorous nanorings and nanohelices have been obtained by placing phosphorene on the surface or inside of carbon nanotubes [39,40], which is a step forward in the preparation of BPNTs. Existing theoretical research shows that BPNTs have excellent and unique physical properties and have potential application prospects in semiconductors [32,34,41,42,43,44,45], sensors [46,47,48,49], new energy [50,51,52], nanoelectromechanical device [53,54], optoelectronics [41,45,55,56], etc. Currently, research on BPNTs primarily centers on structural stability [28,30,31,54,57,58,59,60], mechanical properties [34,53,59,61,62,63], thermal properties [31,54,64,65], and electrical characteristics [34,41,42,43,44,45,56]; however, there has been no in-depth or systematic investigation into its Poisson’s ratio. The Poisson’s ratio characteristics of BPNTs and the underlying potential mechanisms still require further exploration and research by scholars. Among the existing bulk materials with negative Poisson’s ratio, the structures are basically artificially designed to have hinge-like features [1,7,66]. Almost all of the single materials with inherent negative Poisson’s ratio that have been discovered so far are 2D materials [2,13,14,15,16,17,19,20,21,22,23,24,25,27,67,68,69,70]. We are eager to know whether it is possible to obtain 1D materials with negative Poisson’s ratio by curling 2D materials with negative Poisson’s ratio to form nanotubes. If nanotubes with negative Poisson’s ratio can be obtained, the bulk materials with negative Poisson’s ratio, which are different from the structural designs of traditional negative Poisson ratio materials, can be obtained through methods such as stacking and bundling.

In this paper, we have investigated the Poisson’s ratio of BPNTs by using the molecular dynamics (MD) simulation method. The detailed calculations are elaborated in Section 2. In Section 3, the results obtained are presented. It is found that for the armchair black phosphorus nanotubes (ABPNTs), both the radial strain and the wall thickness strain are negatively linearly correlated with the axial strain. The wall thickness strain of the zigzag black phosphorene nanotubes (ZBPNTs) is negatively linearly correlated with the axial strain, while the radial strain has a cubic polynomial function relationship with the axial strain. As the axial strain increases, the radial Poisson’s ratio progressively diminishes from a positive value and becomes negative upon reaching a specific critical axial strain threshold. Finally, a brief conclusion is given in Section 4. This research contributes significantly to the application and development of black phosphorene nanotubes in the semiconductor industry.

## 2. Models and Methods

**Figure 1:**
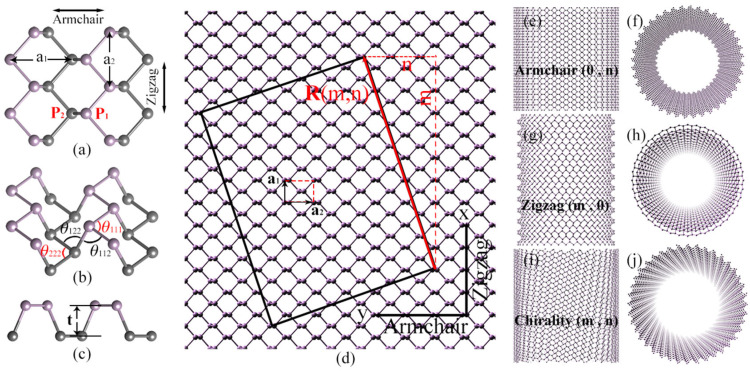
The schematic diagrams of the BPNTs. (**a**) The BP structure, P_1_ and P_2_ are phosphorous atoms on two different surfaces represented by brown and black, respectively. The lattice constants are **a**_1_ = 3.35 Å and **a**_2_ = 4.69 Å. (**b**) The perspective view of BP, the angles θ_111_ = θ_222_ = 97.31° and θ_221_ = θ_112_ = 104.91°. (**c**) The side view of BP, the thickness of a single layer t = 2.12 Å. (**d**) The curling vector R (m, n). (**e**,**f**) ABPNTs and their axial perspective views are shown. (**g**,**h**) ZBPNTs and their axial perspective views are presented. (**i**,**j**) BPNTs with chirality C (m, n) and their corresponding axial perspective views are illustrated.

As shown in Figure 1, BPNTs can be rolled by BP along the vector **R** = m**a**_1_ + n**a**_2_, where m and n are integers representing the number of lattice vectors along the **a**_1_ and **a**_2_ directions, respectively. To distinguish the phosphorous atoms of BPNTs, we label the atoms on the surface of BPNTs as P_1_ and the atoms inside as P_2_, as illustrated in Figure 1a. The chirality of BPNTs is characterized by the integer pair C (m, n). When R aligns with the x-axis (n = 0), we can obtain a zigzag phosphorene nanotube (ZBPNT), denoted as Z(m, 0); when R aligns with the y-axis (m = 0), we can obtain an armchair phosphorene nanotube (ABPNT), denoted as A(0, n). The right side of Figure 1 shows the structural diagrams of BPNTs with different chirality. Both ABPNTs and ZBPNTs possess three types of atomic bonds—Bond_11_ (P_1_ – P_1_), Bond_22_ (P_2_ – P_2_), and Bond_12_ (P_1_ – P_2_)—and four types of bond angles: θ_111_ (∠P_1_P_1_P_1_), θ_222_ (∠P_2_P_2_P_2_), θ_221_, and θ_112_ (∠P_2_P_2_P_1_ and ∠P_1_P_1_P_2_). We constructed models of BPNTs with varying chirality and diameters, specifically A(0, 30), A(0, 60), and A(0, 90) for ABPNTs and Z(30, 0), Z(60, 0), and Z(0, 90) for ZBPNTs. Each BPNT model contains 50 cells along the axial direction. Simulations were performed using the LAMMPS (29 August 2024) [71] open-source software package, employing the Stillinger–Weber (S-W) potential to describe the interactions between phosphorus atoms [69,72,73]. Jiang [73] used the optimized SW potential to calculate the phonon spectrum properties of BP, which are in good agreement with those obtained by ab initio calculations. Meanwhile, the two-body and three-body terms of the SW potential introduce nonlinear effects, enabling the simulation of the nonlinear properties of materials. The SW potential has also been recognized by other scholars and has achieved certain success in the research on the mechanical properties and thermodynamics of BP [69,74,75], making it a powerful tool for current molecular dynamics studies on the properties of BP.

**Figure 2:**
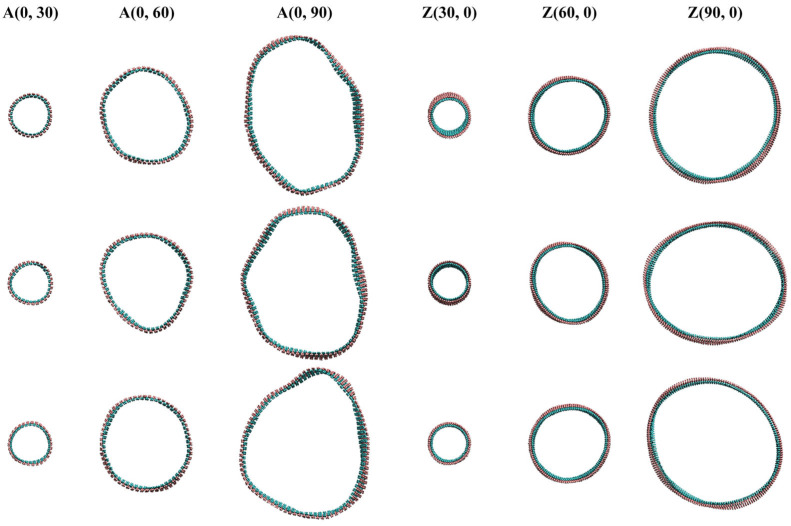
The thermal-induced deformation of the tube wall in BPNTs at room temperature increases with the diameter of the nanotubes.

Figure 2 shows the top views of BPNTs with different radii at the same ambient temperature. It can be seen that the larger the radius of the BPNTs, the more severe the deformation of their tube walls. We utilized the root mean square (RMS) of the atomic distances from the central axis to determine the inner and outer radii of the tube wall. Based on these measurements, we obtained the outer and inner diameter and wall thickness of the BPNTs.

Table 1 shows the inner and outer diameters and the tube wall thickness of several BPNT models with different chirality at an ambient temperature of 300 K. It can be seen from Table 1 that the value of the tube wall thickness does not increase with the increase in the radius of BPNTs. We believe that this error is caused by the deformation of the tube wall as the radius of BPNTs increases. By observing Figure 2, we find that at an ambient temperature of 300 K, as the radius of BPNTs increases, the fluctuating deformation of the tube wall becomes more obvious. In particular, ABPNTs with larger radii have wave-like undulations on their surfaces. Solving for strain can eliminate most of the errors caused by the tube wall deformation. The length strain (ε_l_), radial strain (ε_d_), and thickness strain (ε_t_) of BPNTs are expressed as follows:

ε_l_ = (l − l_0_)/l_0_ (1)

ε_d_ = (d − d_0_)/d_0_ (2)

ε_t_ = (t − t_0_)/t_0_ (3)

In the above formula, l_0_, d_0_, and t_0_ are the length, diameter, and wall thickness of BPNTs without axial deformation, respectively, while l, d, and t correspond to the length, diameter, and wall thickness of BPNTs after axial deformation, respectively. Therefore, we believe that the deformation of the tube wall does not affect the analysis results of the functional relationship between the axial strain and the transverse strain of BPNTs.

Under the NPT ensemble, axial strain (ε_l_) was applied to BPNTs; the ε_l_ range of BPNTs is from −0.2 to 0.4, with an interval of 0.01 between data points. The strain range where BPNTs do not undergo obvious buckling or tensile fracture is selected as the range for our data processing. We do not calculate the relevant data during the continuous change in axial strain. Instead, for each ε_l_ value, after the BPNTs reach the predetermined value of that ε_l_ and undergo sufficient relaxation at the temperature of 300 K. Subsequently, the system was further relaxed under the NVT ensemble to achieve a stable BPNTs structure, and then we calculate the corresponding inner and outer diameters as well as the tube wall thickness. Relaxation under the NVT ensemble was performed at the temperature of 300 K, with a time step of 0.2 fs and a total relaxation duration of 100 ps.

According to the definition, Poisson’s ratio ν = −ε_⊥_/ε_l_, where ε_l_ denotes the longitudinal strain and ε_⊥_ denotes the transverse strain of the material. In continuum mechanics, two distinct conventions are commonly employed for calculating the mechanical properties of tubular structures. Under these conventions, the cross-sectional area of the tube is modeled either as a solid cylinder or as a hollow annular tube. Consequently, different methods for calculating Poisson’s ratio arise based on these two conventions [62,76].

When the nanotube is modeled as a solid cylinder, its radial Poisson’s ratio can be expressed as follows:

ν_d_ = −ε_d_/ε_l_ (4)

When the nanotube is modeled as a hollow tube, its thickness Poisson’s ratio can be expressed as follows:

ν_t_ = −ε_t_/ε_l_ (5)

## 3. Results and Analysis

**Figure 3:**
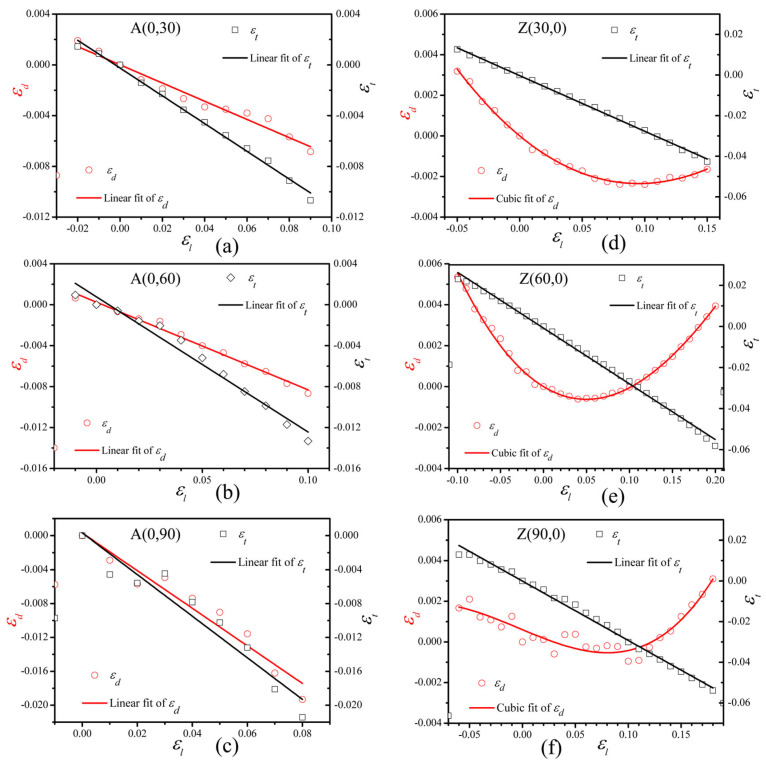
The resultant strain ε_d_ and ε_t_ vs. the applied strain ε_l_ of BPNTs. Red hollow circles represent ε_d_ vs. ε_l_, black hollow squares denote ε_t_ vs. ε_l_. (**a**–**c**) are the strain relationship diagrams of ABPNTs, where the black solid line and red solid line represent the linear fitting results of ε_t_ vs. ε_l_ and ε_d_ vs. ε_l_, respectively. (**d**–**f**) are the strain relationship diagrams of ZBPNTs, where the black solid line is the linear fitting result of ε_t_ vs. ε_l_, and the red solid line is the cubic fitting result of ε_d_ vs. ε_l_.

The ABPNT models, A(0, 30), A(0, 60), and A(0, 90), compressive buckling occurs at axial strains (ε_l_) of −0.03, −0.02, and −0.02, respectively, while tensile fracture occurs at ε_l_ values of 0.10, 0.11, and 0.09, respectively. For the ZBPNT models, Z(30, 0), Z(60, 0), and Z(90, 0), compressive buckling or fracture is observed at ε_l_ values of −0.06, −0.11, and −0.07, respectively, and tensile fracture occurs at ε_l_ values of 0.16, 0.20, and 0.19, respectively. To ensure Poisson’s ratio calculations accurately, we selected a strain range where no significant buckling or fracture occurred in the BPNTs. After obtaining the transverse strains ε_d_ and ε_t_ under different axial strains ε_l_, we can take ε_l_ as the x-axis and ε_d_ and ε_t_ as the y-axis to obtain the function relation graphs of ε_d_-ε_l_ and ε_t_-ε_l_. Figure 3a–c shows the function relation graphs of ε_d_-ε_l_ and ε_t_-ε_l_ for ABPNTs, and Figure 3d–f shows the function relation graphs of ε_d_-ε_l_ and ε_t_-ε_l_ for ZBPNTs.

As illustrated in Figure 3a–c, the scatter plot depicts the relationship between ε_d_-ε_l_ and ε_t_-ε_l_ of ABPNTs. It is evident that ε_d_ and ε_t_ exhibit a predominantly linear correlation with ε_l_. According to Formulas (3) and (4), we obtained the radial Poisson’s ratio (ν_d_) and thickness Poisson’s ratio (ν_t_) of A(0, 30), A(0, 60), and A(0, 90), as summarized in Table 2. Our findings indicate that both ν_d_ and ν_t_ are diameter-dependent, with an increasing trend observed as the diameter of ABPNTs increases.

Figure 3d–f illustrates the scatter plot depicting the relationships among ε_d_, ε_t_, and ε_l_ for ZBPNTs. It is observed that ε_t_ exhibits a predominantly linear correlation with ε_l_, allowing for the determination of the thickness Poisson’s ratio ν_t_ through linear regression analysis. In contrast, ε_d_ does not display a linear relationship with ε_l_. Instead, function fitting reveals that ε_d_ and ε_l_ follow a cubic polynomial relationship. This finding is consistent with the strain characteristics of the thickness observed in BP when stretched along the armchair direction [2] and parallels the strain behavior of graphene in the zigzag direction under similar conditions [14].

The results of the nonlinear fitting of ε_d_-ε_l_ using the function y = −ν_1_x + ν_2_x^2^ + ν_3_x^3^ [2] are summarized in Table 3. According to the definition of ν_d_, where ν_d_ = −∂ε_d_/∂ε_l_, it is derived that ν_d_ = ν_1_ – 2ν_2_ε_l_ – 3ν_3_ε_l_^2^. ν_d_ represents the negative of the slope of the ε_d_-ε_l_ curve. In the initial stage of tensile deformation, ν_d_ is positive, and as the tensile strain increases, ν_d_ transitions to negative values, which means that the outer diameter of the nanotube begins to expand. In Figure 3d–f, the negative Poisson’s ratio corresponds to the stage where the red solid line increases in the opposite direction after the minimum value of ε_d_. For Z(60,0) and Z(90,0), their ε_d_ can even be positive, which means that after a process of first contracting ann then expanding, their outer diameters can exceed their initial values.

Table 4 presents a comparative analysis of the Poisson’s ratios of BPNTs obtained through various methods. The thickness Poisson’s ratios (ν_t_) are basically close to the results of other scholars, while the radial Poisson’s ratios (ν_d_) yield divergent results. In our view, the reason for the differences in ν_d_ observed in ZBPNTs is that some scholars, based on elastic theory, treat the deformation of BPNTs as a type of harmonic oscillator. They solve the relevant elastic coefficients and Poisson’s ratio under the presupposition of linear elastic deformation. The obtained Poisson’s ratio values either correspond to the specific ε_l_ at that time or are the expected values E(ν_d_) within a certain deformation range. In their data processing, the corresponding functional relationship diagram between radial strain and axial strain is not provided. Therefore, in the calculation of the radial Poisson’s ratio of ZBPNTs, the nonlinear radial Poisson’s ratio fails to be presented.

## 4. Conclusions

In this study, molecular dynamics simulations were employed to investigate the Poisson’s ratios of armchair black phosphorene nanotubes (ABPNTs) and zigzag black phosphorene nanotubes (ZBPNTs). The results indicate that the radial Poisson’s ratio ν_d_ of ABPNTs is positively correlated with the tube diameter. Specifically, as the diameter increases, ν_d_ also increases. In our analysis, the ν_d_ values for A(0, 30), A(0, 60), and A(0, 90) were 0.072, 0.086, and 0.222, respectively. Similarly, the thickness Poisson’s ratio ν_t_ of ABPNTs exhibits a positive correlation with the diameter, with ν_t_ values of 0.110, 0.132, and 0.246 for A(0, 30), A(0, 60), and A(0, 90), respectively. Considering the influence of tube wall deformation, we estimate that in the absence of significant wall deformation, the ν_d_ value of ABPNTs is approximately 0.08, while the ν_t_ value is around 0.12.

For ZBPNTs, the thickness Poisson’s ratio ν_t_ does not exhibit a significant dependence on the diameter. In our study, the ν_t_ values for Z(30, 0), Z(60, 0), and Z(90, 0) were 0.274, 0.271, and 0.292, respectively. The relationship between the radial strain ε_d_ and axial strain ε_l_ in ZBPNTs is nonlinear and can be described by a cubic function: y = −ν_1_x + ν_2_x^2^ + ν_3_x^3^, where ν_1_, ν_2_, and ν_3_ represent the first-order, second-order, and third-order Poisson’s ratios, respectively. The radial Poisson’s ratio ν_d_ of ZBPNTs varies with axial strain ε_l_. As ZBPNTs are stretched and ε_l_ increases, ν_d_ decreases from a positive value to a negative one, leading to an inverse expansion behavior. When ε_l_ reaches a critical value, ε_d_ may become positive, and the diameter of ZBPNTs exceeds its initial size, resulting in uniform radial expansion.

Our findings provide valuable insights into the design and application of materials with negative Poisson’s ratios. The unique tensile expansion effect observed in ZBPNTs offers potential for developing novel negative Poisson’s ratio composite materials.

## Figures and Tables

**Table 1:** The inner and outer diameters and wall thickness of BPNTs (Å).

	A(0, 30)	A(0, 60)	A(0, 90)	Z(30, 0)	Z(60, 0)	Z(90, 0)
D_out_	43.93	85.29	124.98	34.35	65.69	97.10
D_in_	39.71	81.11	120.88	30.15	64.7	92.90
t	2.11	2.09	2.05	2.10	2.11	2.10

**Table 2:** The radial Poisson’s ratio (ν_d_) and thickness Poisson’s ratio (ν_t_) of ABPNTs.

	A(0, 30)	A(0, 60)	A(0, 90)
ν_d_	0.072	0.086	0.222
ν_t_	0.110	0.132	0.246

**Table 3:** The radial Poisson’s ratio (ν_d_) and thickness Poisson’s ratio (ν_t_) of ZBPNTs.

	Z(0, 30)	Z(0, 60)	Z(0, 90)
ν_1_	0.051	0.026	0.022
ν_2_	0.291	0.275	0.01
ν_3_	−0.161	−0.244	1.038
ν_t_	0.274	0.271	0.292

**Table 4:** A comparative analysis of the results obtained from diverse studies.

Researcher	Method and Package	ABPNTs(ν_d_)	ZBPNTs (ν_1_/ν_d_)	ABPNTs(ν_t_)	ZBPNTs(ν_t_)
This paper	MD (LAMMPS)	0.072–0.222	−0.055~0.088	0.110–0.246	0.274–0.292
Chen et al. [62]	Force field (COMPASS)	--	--	0.45	0.11
Sorkin et al. [76]	Tight binding	0.47	0.07	0.11	0.21
Ansari et al. [77]	DFT-FEM	--	--	0.47	--

## Data Availability

Data are contained within the article.

## References

[1] Lakes R. (1987). Foam Structures with a Negative Poisson’s Ratio. Science.

[2] Jiang J.-W., Park H.S. (2014). Negative poisson’s ratio in single-layer black phosphorus. Nat. Commun..

[3] Jiang J.-W., Kim S.Y., Park H.S. (2016). Auxetic nanomaterials: Recent progress and future development. Appl. Phys. Rev..

[4] Borcea C.S., Ileana S. (2018). Periodic Auxetics: Structure and Design. Q. J. Mech. Appl. Math..

[5] Ren X., Das R., Tran P., Ngo T.D., Xie Y.M. (2018). Auxetic metamaterials and structures: A review. Smart Mater. Struct..

[6] Gan Z., Zhuge Y., Thambiratnam D.P., Chan T.H., Zahra T., Asad M. (2022). Recent advances in auxetics: Applications in cementitious composites. Int. J. Prot. Struct..

[7] Shukla S., Behera B. (2022). Auxetic fibrous structures and their composites: A review. Compos. Struct..

[8] Dong S., Hu H. (2023). Sensors Based on Auxetic Materials and Structures: A Review. Materials.

[9] Shukla S., Behera B.K. (2022). Auxetic fibrous materials and structures in medical engineering—A review. J. Text. Inst..

[10] Lv Y., Zhang Y., Gong N., Li Z.X., Lu G., Xiang X. (2019). On the out-of-Plane Compression of a Miura-Ori Patterned Sheet. Int. J. Mech. Sci..

[11] Zhang J., Lu G., You Z. (2020). Large deformation and energy absorption of additively manufactured auxetic materials and structures: A review. Compos. Part B Eng..

[12] Zhang J., Lu G., Wang Z., Ruan D., Alomarah A., Durandet Y. (2018). Large deformation of an auxetic structure in tension: Experiments and finite element analysis. Compos. Struct..

[13] Du Y., Maassen J., Wu W., Luo Z., Xu X., Ye P.D. (2016). Auxetic Black Phosphorus: A 2D Material with Negative Poisson’s Ratio. Nano Lett..

[14] Jiang J.-W., Chang T., Guo X., Park H.S. (2016). Intrinsic Negative Poisson’s Ratio for Single-Layer Graphene. Nano Lett..

[15] Jiang J.-W., Park H.S. (2016). Negative Poisson’s Ratio in Single-Layer Graphene Ribbons. Nano Lett..

[16] Wan J., Jiang J.-W., Park H.S. (2017). Negative Poisson’s ratio in graphene oxide. Nanoscale.

[17] Yuan K., Li M.-Y., Liu Y.-Z., Li R.-Z. (2019). Design and Prediction of a Novel Two-Dimensional Carbon Nanostructure with In-Plane Negative Poisson’s Ratio. J. Nanomater..

[18] Zhao S., Hu H., Kamrul H., Chang Y., Zhang M. (2019). Development of auxetic warp knitted fabrics based on reentrant geometry. Text. Res. J..

[19] Qin G., Qin Z. (2020). Negative Poisson’s ratio in two-dimensional honeycomb structures. npj Comput. Mater..

[20] Zhang C., Lu C., Pei L., Li J., Wang R. (2019). Molecular Dynamics Simulation of the Negative Poisson’s Ratio in Graphene/Cu Nanolayered Composites: Implications for Scaffold Design and Telecommunication Cables. ACS Appl. Nano Mater..

[21] Sun M., Schwingenschlögl U. (2021). Unique Omnidirectional Negative Poisson’s Ratio in δ-Phase Carbon Monochalcogenides. J. Phys. Chem. C.

[22] Ho V.H., Ho D.T., Nguyen C.T., Kim S.Y. (2022). Negative out-of-plane Poisson’s ratio of bilayer graphane. Nanotechnology.

[23] Wang Y., Yu L., Zhang F., Chen Q., Zhan Y., Meng L., Zheng X., Wang H., Qin Z., Qin G. (2022). The consistent behavior of negative Poisson’s ratio with interlayer interactions. Mater. Adv..

[24] Yu L., Wang Y., Zheng X., Wang H., Qin Z., Qin G. (2022). Emerging negative Poisson’s ratio driven by strong intralayer interaction response in rectangular transition metal chalcogenides. Appl. Surf. Sci..

[25] Zhu Y., Cao X., Tan Y., Wang Y., Hu J., Li B., Chen Z. (2023). Single-Layer MoS2: A Two-Dimensional Material with Negative Poisson’s Ratio. Coatings.

[26] Ren K., Ma X., Liu X., Xu Y., Huo W., Li W., Zhang G. (2022). Prediction of 2D IV–VI semiconductors: Auxetic materials with direct bandgap and strong optical absorption. Nanoscale.

[27] Wen Y., Gao E., Hu Z., Xu T., Lu H., Xu Z., Li C. (2019). Chemically modified graphene films with tunable negative Poisson’s ratios. Nat. Commun..

[28] Guan J., Zhu Z., Tománek D. (2014). High Stability of Faceted Nanotubes and Fullerenes of Multiphase Layered Phosphorus: A Computational Study. Phys. Rev. Lett..

[29] Guo H., Lu N., Dai J., Wu X., Zeng X.C. (2014). Phosphorene Nanoribbons, Phosphorus Nanotubes, and Van Der Waals Multilayers. J. Phys. Chem. C.

[30] Shahnazari A., Ansari R., Rouhi S. (2017). On the stability characteristics of zigzag phosphorene nanotubes: A finite element investigation. J. Alloy Compd..

[31] Cai K., Wan J., Wei N., Cai H., Qin Q.-H. (2016). Thermal stability of a free nanotube from single-layer black phosphorus. Nanotechnology.

[32] Fernández-Escamilla H.N., Quijano-Briones J.J., Tlahuice-Flores A. (2016). Chiral phosphorus nanotubes: Structure, bonding, and electronic properties. Phys. Chem. Chem. Phys..

[33] Seifert G., Hernández E. (2000). Theoretical prediction of phosphorus nanotubes. Chem. Phys. Lett..

[34] Zhang W., Yin J., Zhang P., Ding Y. (2017). Strain/stress engineering on the mechanical and electronic properties of phosphorene nanosheets and nanotubes. RSC Adv..

[35] Shi J., Cai K., Liu L.-N., Qin Q.-H. (2017). Self-assembly of a parallelogram black phosphorus ribbon into a nanotube. Sci. Rep..

[36] Cai K., Liu L., Shi J., Qin Q.H. (2017). Winding a nanotube from black phosphorus nanoribbon onto a CNT at low temperature: A molecular dynamics study. Mater. Des..

[37] Cai K., Shi J., Liu L., Qin Q. (2017). Fabrication of an ideal nanoring from a black phosphorus nanoribbon upon movable bundling carbon nanotubes. Nanotechnology.

[38] Cai K., Liu L., Shi J., Qin Q.H. (2017). Self-Assembly of a Jammed Black Phosphorus Nanoribbon on a Fixed Carbon Nanotube. J. Phys. Chem. C.

[39] Zhang J., Zhao D., Xiao D., Ma C., Du H., Li X., Zhang L., Huang J., Huang H., Jia C. (2017). Assembly of Ring-Shaped Phosphorus within Carbon Nanotube Nanoreactors. Angew. Chem. Int. Ed. Engl..

[40] Choi Y.W., Lee Y., Kim K., Zettl A., Cohen M.L. (2025). Atomic and Electronic Structures of 1D Phosphorus Nanoring and Nanohelix. ACS Nano.

[41] Badehian H.A., Gharbavi K. (2021). Effect of silicon doping on the electronic and optical properties of phosphorous nanotubes. Optik.

[42] Fernández-Escamilla H.N., Guerrero-Sanchez J., Martínez-Guerra E., Takeuchi N. (2019). Structural and Electronic Properties of Double-Walled Black Phosphorene Nanotubes: A Density Functional Theory Study. J. Phys. Chem. C.

[43] Dai X., Zhang L., Wang Z., Li J., Li H. (2019). Effect of C and O dopant atoms on the electronic properties of black phosphorus nanotubes. Comput. Mater. Sci..

[44] Li Q., Wang H., Yang C., Li Q., Rao W. (2018). Theoretical prediction of high carrier mobility in single-walled black phosphorus nanotubes. Appl. Surf. Sci..

[45] Li C., Xie Z., Chen Z., Cheng N., Wang J., Zhu G. (2018). Tunable Bandgap and Optical Properties of Black Phosphorene Nanotubes. Materials.

[46] Ou P., Zhou X., Li X.-Y., Chen Y., Chen C., Meng F., Song J. (2022). Single-Walled Black Phosphorus Nanotube as a NO2 Gas Sensor. Mater. Today Commun..

[47] Maria J.P., Nagarajan V., Chandiramouli R. (2021). Chemosensing nature of black phosphorene nanotube towards C14H9Cl5 and C10H5Cl7 molecules – A first-principles insight. Comput. Theor. Chem..

[48] Kazerooni H.B., Ghayour R., Pesaran F. (2021). Analysis and application of zigzag phosphorene nanotube as gas nanosensor. Appl. Phys. A.

[49] Saravanan S., Nagarajan V., Chandiramouli R. (2019). Adsorption insights of amine vapors on black phosphorene nanotubes—A first-principles study. Mater. Res. Express.

[50] Cao J., Shi J., Hu Y., Wu M., Ouyang C., Xu B. (2017). Lithium ion adsorption and diffusion on black phosphorene nanotube: A first-principles study. Appl. Surf. Sci..

[51] Zhang W., Cui Y., Zhu C., Huang B., Yan S., Lou Y., Zhang P. (2023). Sequential hydrogen storage in phosphorene nanotubes: A molecular dynamics study. Int. J. Hydrogen Energy.

[52] Wang H., Gao Q., Liu C., Cao Y., Liu S., Zhang B., Hu Z., Sun J. (2022). Anisotropic black phosphorene nanotube anodes afford ultrafast kinetic rate or extra capacities for Li-ion batteries. Chin. Chem. Lett..

[53] Tran K., Taylor P.D., Spencer M.J.S. (2024). Electromechanical strain response of phosphorene nanotubes. J. Phys. Mater..

[54] Cuba-Supanta G., Fernández-Escamilla H.N., Guerrero-Sanchez J., Rojas-Tapia J., Takeuchi N. (2020). Structural properties and thermal stability of multi-walled black phosphorene nanotubes and their operation as temperature driven nanorotors. Nanoscale.

[55] Guan L., Chen G., Tao J. (2016). Prediction of the electronic structure of single-walled black phosphorus nanotubes. Phys. Chem. Chem. Phys..

[56] Yang J., Xie Y., Wang S., Jiang N., Chen L., Wang X., Zhang J. (2022). Manipulating the electronic, magnetic, and optical properties of single-walled armchair phosphorus nanotubes by encapsulating transition metal nanowires. Mater. Today Commun..

[57] Liu P., Pei Q.-X., Huang W., Zhang Y.-W. (2017). Mechanical properties and fracture behaviour of defective phosphorene nanotubes under uniaxial tension. J. Phys. D Appl. Phys..

[58] Nguyen V.-T. (2018). Effects of Missing Atom Defect on the Mechanical Properties of Black Phosphorene Nanotube. Int. J. Eng. Res. Appl..

[59] Sorkin V., Zhang Y.-W. (2018). Effect of vacancies on the mechanical properties of phosphorene nanotubes. Nanotechnology.

[60] Liu P., Pei Q.-X., Huang W., Zhang Y.-W. (2018). Strength and buckling behavior of defective phosphorene nanotubes under axial compression. J. Mater. Sci..

[61] Ansari R., Shahnazari A., Rouhi S. (2017). A density-functional-theory-based finite element model to study the mechanical properties of zigzag phosphorene nanotubes. Phys. E Low-Dimens. Syst. Nanostructures.

[62] Chen W.-H., Yu C.-F., Chen I.-C., Cheng H.-C. (2017). Mechanical property assessment of black phosphorene nanotube using molecular dynamics simulation. Comput. Mater. Sci..

[63] Hao J., Wang Z., Peng Y., Wang Y. (2017). Structure and elastic properties of black phosphorus nanotubes: A first-principles study. Phys. Status solidi (b).

[64] Wang L., Wang C. (2018). Negative/zero thermal expansion in black phosphorus nanotubes. Phys. Chem. Chem. Phys..

[65] Hao F., Liao X., Xiao H., Chen X. (2016). Thermal conductivity of armchair black phosphorus nanotubes: A molecular dynamics study. Nanotechnology.

[66] Lakes R.S. (2017). Negative-Poisson’s-Ratio Materials: Auxetic Solids. Annu. Rev. Mater. Res..

[67] Yuan K., Zhao Y., Li M., Liu Y. (2021). Predicting an ideal 2D carbon nanostructure with negative Poisson’s ratio from first principles: Implications for nanomechanical devices. Carbon Lett..

[68] Gao Y., Wen M., Zhang X., Wu F., Xia Q., Wu H., Dong H. (2021). Factors affecting the negative Poisson’s ratio of black phosphorus and black arsenic: Electronic effects. Phys. Chem. Chem. Phys..

[69] Jiang J.-W., Rabczuk T., Park H.S. (2015). A Stillinger–Weber potential for single-layered black phosphorus, and the importance of cross-pucker interactions for a negative Poisson’s ratio and edge stress-induced bending. Nanoscale.

[70] Ho D.T., Park S.D., Kwon S.Y., Park K., Kim S.Y. (2014). Negative Poisson’s Ratios in Metal Nanoplates. Nat. Commun..

[71] Plimpton S. (1995). Fast Parallel Algorithms for Short-Range Molecular Dynamics. J. Comput. Phys..

[72] Jiang J.-W. (2017). An empirical description for the hinge-like mechanism in single-layer black phosphorus: The angle–angle cross interaction. Acta Mech. Solida Sin..

[73] Jiang J.-W. (2015). Parametrization of Stillinger–Weber potential based on valence force field model: Application to single-layer MoS_2_and black phosphorus. Nanotechnology.

[74] Sha Z.-D., Pei Q.-X., Ding Z., Jiang J.-W., Zhang Y.-W. (2015). Mechanical properties and fracture behavior of single-layer phosphorene at finite temperatures. J. Phys. D Appl. Phys..

[75] Yang Z., Zhao J., Wei N. (2015). Temperature-dependent mechanical properties of monolayer black phosphorus by molecular dynamics simulations. Appl. Phys. Lett..

[76] Sorkin V., Zhang Y.W. (2016). Mechanical properties of phosphorene nanotubes: A density functional tight-binding study. Nanotechnology.

[77] Ansari R., Aghdasi P., Shahnazari A. (2024). A comprehensive study of mechanical properties in armchair phosphorene nanotubes using DFT-based finite element analysis. Appl. Phys. A.

